# News and Coming Events

**Published:** 1908-01-11

**Authors:** 


### NEWS AND COMING EVENTS.

It has been decided to build a new general hospital for Lewes.

Under the will of Mr. Isaac Coley, of Peckham, S.E., Guy's Hospital, St. Thomas's Hospital, University College Hospital, and the Lying-in Hospital will each benefit by a sum of about £3,000.

The insufficient accommodation at the Ulster Hospital for Women and Children at Mount Pottinger greatly hampers the work of that institution. It has been decided accordingly to rebuild the hospital on its present site at a cost of £10,000.

A case of suicidal poisoning by eating yew leaves, the first on record in England, occurred at Devonport recently. The deceased, a man named Attwell, had chewed and swallowed a large quantity of yew leaves, and was found in a state of collapse thirty yards from the place where yew trees were growing.

"What did Mozart die of?" This question, which has some interest for students of the great musician, Dr. Barraud tries to answer in an exhaustive paper, which is, indeed, a thesis on Mozart. According to Dr. Barraud, Mozart died of albuminuria and nephritis, and not, as is usually stated, of tubercular meningitis.

The next meeting of the Society for the Study of Inebriety will be held in the rooms of the Medical Society, 11 Chandos Street, Cavendish Square, W., on Tuesday, January 14, next, at 4 p.m., when Mr. W. Edwards, F.C.S., will open a discussion on "The Teaching of Hygiene and Temperance in Schools."

The death is announced of Sir Alfred Baring Garrod, F.R.S., one of the consulting physicians to King's College Hospital. Sir Alfred Garrod, who was born in 1819, was one of the oldest physicians on the register, and was Vice-President of the Royal College of Physicians in 1888, having been knighted in the previous year.

A special law has been passed in Austria to control and regulate radiotherapy. It provides special restrictions for x-ray specialists, who are required to furnish detailed reports of all their cases and to be specially licensed.

A new wing, consisting of a large ward with eight beds and two cots and the usual additionel rooms, has been opened at the Tynemouth Victoria Jubilee Infirmary. The whole cost of the building and its furnishing, amounting to over £1,400, has been defrayed by Mrs. T. C. Leitch, in memory of her husband, who was the first Town Clerk of the Borough of Tynemouth.

The opening lecture of the Mount Vernon Hospital post-graduate course was given by Sir Thomas Clifford Allbutt, F.R.S., consulting physician to the hospital, in the lecture-room at the central out-patient department, 7 Fitzroy Square, London, W., on Thursday, January 9, at 5 p.m. Professor Allbutt took as his subject "Angina Pectoris."

On December 23 last a presentation was made at the Royal College of Surgeons to Mr. C. R. Hewitt, who for the past twenty-two years has held the post of assistant in the College library. The subscribers numbered some seventy-five friends, on whose behalf Professor William Osler, F.R.S., handed to Mr. Hewitt a piece of plate, suitably inscribed, and a cheque. Professor Osler and Mr. Henry T. Butlin eulogised Mr. Hewitt's good work, and wished him success in his new post at the Royal Society of Medicine.

The second annual dinner of the past and present students of the Royal London Ophthalmic Hospital will take place at the Trocadero Restaurant, Shaftesbury Avenue, W., on Wednesday, January 29, at 7.45 for 8 p.m., with Sir John Tweedy, consulting surgeon to the hospital, in the chair. Each student is entitled to introduce two guests, and tickets (price 10s. 6d. each, exclusive of wine) may be had on application to either of the Honorary Secretaries, Mr. Arnold Lawson, 12 Harley Street, London, W., and Mr. J. Herbert Parsons, 27 Wimpole Street, London, W.

